# Supplementary figures and images for: Modeling and Molecular Dynamics of the 3D Structure of the HPV16 E7 Protein and Its Variants
Source: Int J Mol Sci. 2021 Jan 30;22(3):1400. doi: 10.3390/ijms22031400 (PMC7866783; doi:10.3390/ijms22031400)

# \figure

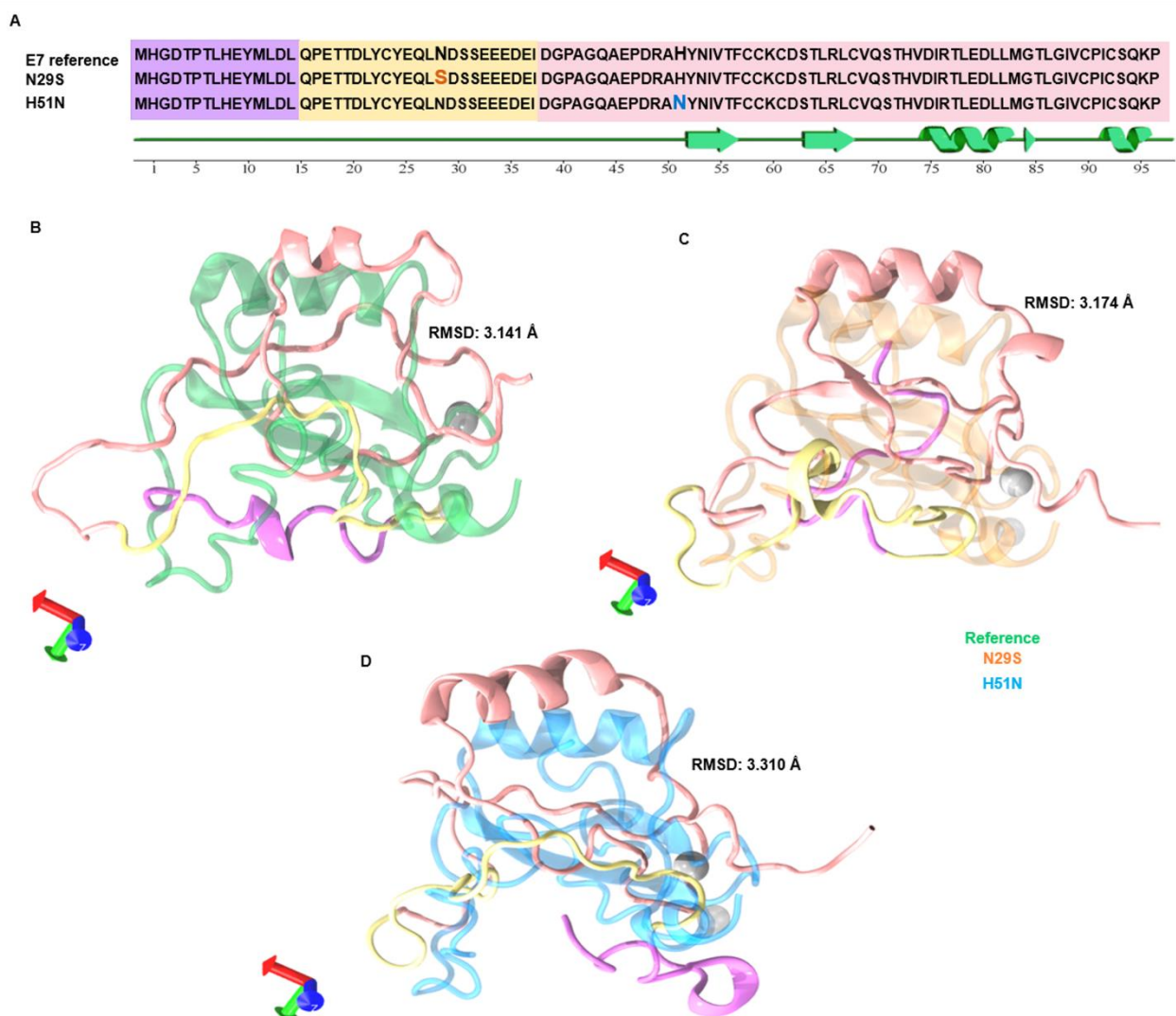

Figure S1

Supplement: Supplementary file 1 [file ijms-22-01400-s001.pdf]
